# Genome-wide identification, characterization and expression analysis of MATE family genes in apple (*Malus* × *domestica* Borkh)

**DOI:** 10.1186/s12864-021-07943-1

**Published:** 2021-08-30

**Authors:** Weihan Zhang, Liao Liao, Jinsheng Xu, Yuepeng Han, Li Li

**Affiliations:** 1 Hubei Key Laboratory of Agricultural Bioinformatics, College of Informatics, Huazhong Agricultural University, Wuhan, 430070 People’s Republic of China; 2 CAS Key Laboratory of Plant Germplasm Enhancement and Specialty Agriculture, Wuhan Botanical Garden, The Innovative Academy of Seed Design, Chinese Academy of Sciences, Wuhan, 430074 People’s Republic of China; 3 Hubei Hongshan Laboratory, Huazhong Agricultural University, Wuhan, 430070 People’s Republic of China

**Keywords:** Apple, MATE, Gene family, Expression profile, Disease resistance

## Abstract

**Background:**

As an important group of the multidrug efflux transporter family, the multidrug and toxic compound extrusion (MATE) family has a wide range of functions and is distributed in all kingdoms of living organisms. However, only two MATE genes in apple have been analyzed and genome-wide comprehensive analysis of MATE family is needed.

**Results:**

In this study, a total of 66 MATE (*MdMATE*) candidates encoding putative MATE transporters were identified in the apple genome. These *MdMATE* genes were classified into four groups by phylogenetic analysis with MATE genes in *Arabidopsis*. Synteny analysis reveals that whole genome duplication (WGD) and segmental duplication events played a major role in the expansion of MATE gene family in apple. *MdMATE* genes show diverse expression patterns in different tissues/organs and developmental stages. Analysis of *cis*-regulatory elements in *MdMATE* promoter regions indicates that the function of *MdMATE* genes is mainly related to stress response. Besides, the changes of gene expression levels upon different pathogen infections reveal that *MdMATE* genes are involved in biotic stress response.

**Conclusions:**

In this work, we systematically identified *MdMATE* genes in apple genome using a set of bioinformatics approaches. Our comprehensive analysis provided valuable resources for improving disease resistance in apple and further functional characterization of MATE genes in other species.

**Supplementary Information:**

The online version contains supplementary material available at 10.1186/s12864-021-07943-1.

## Background

The multidrug and toxic compound extrusion (MATE) protein belongs to a multidrug efflux transporter family, which plays a role in transporting multiple kinds of substrates, such as secondary metabolites and phytohormones [1]. The process of detoxification mainly involves four transporter families: ATP-binding cassette superfamily (ABC), resistance/nodulation/division family (RND), small gene multidrug resistance family (SMR), and major facilitator superfamily (MFS) [2]. Members of the ABC superfamily are considered to be primary transporters, and ATP provides energy during transport [3]. The transmembrane transport of primary transporters is mainly carried out by releasing energy [4]. The members of RND, SMR and MFS are secondary transporters and mainly use the electrochemical penetration potential caused by the difference between the internal and external material concentration of the membrane to implement transport process [4]. In these secondary transporter families, numerous MFS family members with different functions are widely distributed in both higher and lower organisms. Most MFS superfamily members contained 12 membrane alpha-helix and use cations (H^+^ or Na^+^ ions) electrochemical gradients to drive substrate export [5, 6]. In 1998, a new multidrug efflux system named NorM that can increase the efflux of norfloxacin in the absence of a multidrug efflux system in the host was found in *E. coli*. Meanwhile, a high sequence homology multidrug efflux protein was also found and named YdhE [7]. Although the structure of NorM is similar to the MFS superfamily that has 12 transmembrane regions (TMs), both NorM and YdhE proteins have no homology with members of the MFS superfamily, nor a specific signal sequence for this family [8]. Therefore, the new secondary transporter family, NorM and YdhE, become the fifth kind of transporter family which is named as multidrug and toxic compound extrusion (MATE) [9].

Previous study has shown that MATE transporters are widely distributed in all kingdoms of living organisms [1]. The protein length of most MATE transporters is 400–700 amino acids, containing 8—12 transmembrane domains [10, 11]. The first plant MATE transporter was identified in *Arabidopsis* named *A. thaliana aberrant lateral root formation 5* (*AtALF5*), which is associated with roots epidermal cells development and toxic compounds export [12]. In recent years, several studies have been conducted to characterize the function of MATE proteins in the model organism *Arabidopsis* and have shown that MATE proteins have various functions. For example, the *TRANSPARENT TESTA 12* (*TT12*) encodes a MATE transporter which can mediate anthocyanin transport [13] and the *AtDTX1* (*DETOXIFICATION 1*) is the first multi-specific MATE transporter control the export of toxic compounds from the cytoplasm [10]. In contrast to the mammal, plant genomes carry a larger number of MATE genes [14]. For instance, 56 in *Arabidopsis* [10], 48 in potato [6], 49 in maize [15], 45 in rice [16] and 117 in soybean [17]. Extensive research on MATE gene family suggest diverse functions, which are involved in stress responses, secondary metabolite and phytohormone transport, plant growth and development [18, 19]. However, the function and status of MATE gene family in fruit trees have rarely been reported.

Apple (*Malus × domestica* Borkh.) is one of the most economically important and popular perennial fruit crops in temperate regions. It is highly susceptible to infection by pathogen and greatly affect quality and yield. The development of resistance in pathogens and the large investment of farmers in fungicides have become an important issue [20]. A previous study showed that the expression levels of MATE gene family are involved in pathogen susceptibility [21]. However, as far as we know only two *MATE* genes (*MdMATE1* and *MdMATE2*) in apple were analyzed, which are homologs of *TT12* in *Arabidopsis* and participate in proanthocyanidins accumulating in cells and flavonoid transport [22]. A comprehensive analysis of the MATE gene family is needed in the apple genome.

In this study, we identified 66 *MATE* genes (*MdMATE*) in apple genome and conducted comprehensive analysis regarding their phylogenetic relationship, synteny, gene structure, evolution, expression in different tissues/organs and developmental stages, and *cis*-elements. Synteny analysis shows that WGD/segmental duplication events played a major role in the expansion of MATE gene family in apple. Expression analysis suggests that *MdMATE* genes have specific expression patterns in different tissues/organs and developmental stages. Correlation analysis reveals that the functions of duplicated gene pairs may have divergence. We also found that *MdMATE* genes are involved in biotic stress response. Our study provides clues for further functional studies of MATE genes in plants and improvement of disease resistance in apple.

## Results

### Genome-wide identification of MATE transporters in apple

A total of 66 genes encoding MATE transporters were identified in the apple reference genome GDDH13 version 1.1 [23] after homologous sequence alignment with *AtMATE* genes from *A. thaliana* and manual filtering (see Methods) (Additional file 1: Table S1). Since two MATE transporters, MdMATE1 and MdMATE2, have been reported in previous study [22], we renamed the other 64 transporters as MdMATE3 – MdMATE66 based on their physical locations (Additional file 1: Table S2).

The basic properties including the length of protein sequence, theoretical isoelectric point (pI), molecular weight (MW) and subcellular localization were analyzed to further characterize the MdMATE proteins (Additional file 1: Table S2). The 66 MdMATE proteins consist of 406 to 712 amino acids in length, which is quite similar to MATE proteins in *Arabidopsis* (400—700 amino acids) [10], but different from soybean (80–593 amino acids) [17], *Populus* (120–608 amino acids) [24] and rice (469—575 amino acids) [16]. The MW and pI ranges from 45.20 to 77.02 kDA and 5.05 to 9.54, respectively. Subcellular location prediction results include plasma membrane (56), chloroplast (5), cytoplasm (2), vacuole (2), and endoplasmic reticulum (1).

### Phylogenetic, gene structures and motif composition analysis of the MATE family in apple

**Fig. 1:**
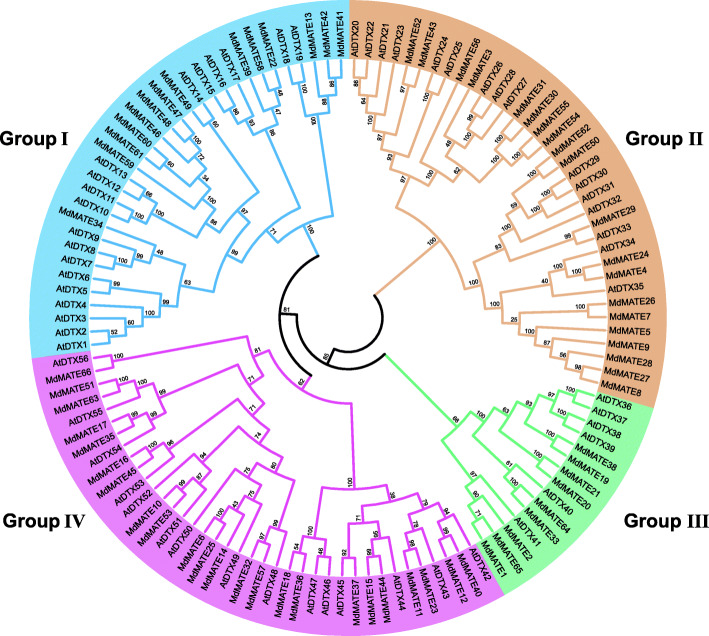
The unrooted maximum likelihood phylogenetic tree of MATE family members in apple and *Arabidopsis*. The different colors indicate different groups (Group I in blue, Group II in orange, Group III in green and Group IV in pink). ‘MdMATE’ represents MATE members from apple, ‘AtDTX’ represents MATE members from *Arabidopsis*. Numbers on the nodes are bootstrap values in percentage (1000 replicates)

**Fig. 2:**
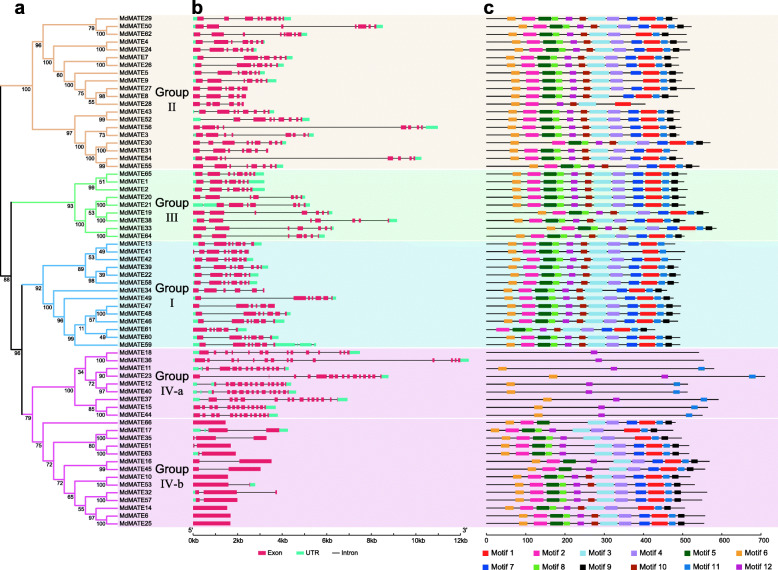
Phylogenetic relationships and structures of 66 *MdMATE* genes. **A**. A Phylogenetic tree inferred from full length sequence of *MdMATE* genes. The number on the nodes are bootstrap value in percentage (1000 replicates). Color of branches represents different groups same with the phylogenetic tree in Fig. 1 (Group I in blue, Group II in orange, Group III in green and Group IV in pink). **B**. Exon-intron structure of *MdMATE* genes. The boxes represent exons or UTRs, lines represent introns. **C**. Motif composition of MdMATE proteins. Different color boxes indicate different motifs. The length of genes or proteins can be estimated using the scale at the bottom

Using the full-length protein sequences of the 122 MATE transporters, including 56 from *Arabidopsis* and 66 identified in apple, we constructed a maximum likelihood (ML) phylogenetic tree (Fig. 1). The 66 MdMATE transporters were divided into four groups (I, II, III and IV), which is in accordance with what was reported for MATE transports in *Arabidopsis* [10]. A neighbor-joining (NJ) tree was also constructed to validate the phylogenetic relationship and show a similar grouping mode as ML tree (Additional file 2: Fig. S1). The group sizes vary from 9 to 23. Group I, II, III and IV contain 14, 20, 9 and 23 MdMATE transporters, respectively. We further aligned MdMATE transporters to construct an individual phylogenetic tree by the same method and parameters and got consistent clustering patterns (Fig. 2A).

To gain more insight into the diversity of the MATE family in apple, we examined the exon-intron organization of all the identified *MdMATE* genes (Fig. 2B). The *MdMATE* genes display one to fifteen exons (5 with one exon, 7 with two exons, 2 with three exons, one with five exons, 5 with six exons, 24 with seven exons, 11 with eight exons, and 11 with nine or more exons). The length of exons ranging from 9 (*MdMATE3*) to 1680 (*MdMATE6*) bp. The fewest number of exons are observed in *MdMATE6*, *MdMATE25*, *MdMATE14*, *MdMATE10*, and *MdMATE66.* These five genes all have only one exon and clustered in group IV. Additionally, 9 genes have more than thirteen exons. Overall, MATE gene family in apple showed complex gene structures with varying exons and lengths.

Motifs of the 66 MdMATE proteins were identified by MEME [25] to analyze the function and/or structural roles of highly conserved amino acid residues in active proteins [26]. A total of 12 conserved motifs, designated as motif 1 to motif 12, were identified (Fig. 2C and Additional file 2: Fig. S2). Most MdMATE proteins have similar motifs within the same group. Of these, motif 12 was found in all the MdMATE proteins except for *MdMATE66*. Interestingly, in group IV, some MdMATE proteins have fewer motifs (≤3), but more exons (≥13) than other genes. Based on the structure and motif differences in group IV, the group IV we further divided into two subgroups: Group IV-a and Group IV-b (Fig. 2A). The group IV-a with more exons but fewer motifs, while the group IV-b is in contrast. These observations are consistent with studies of MATE gene family in other species such as soybean [17], cotton [27] and flax [28].

### Chromosomal location and evolution of apple MATE transporters

**Fig. 3:**
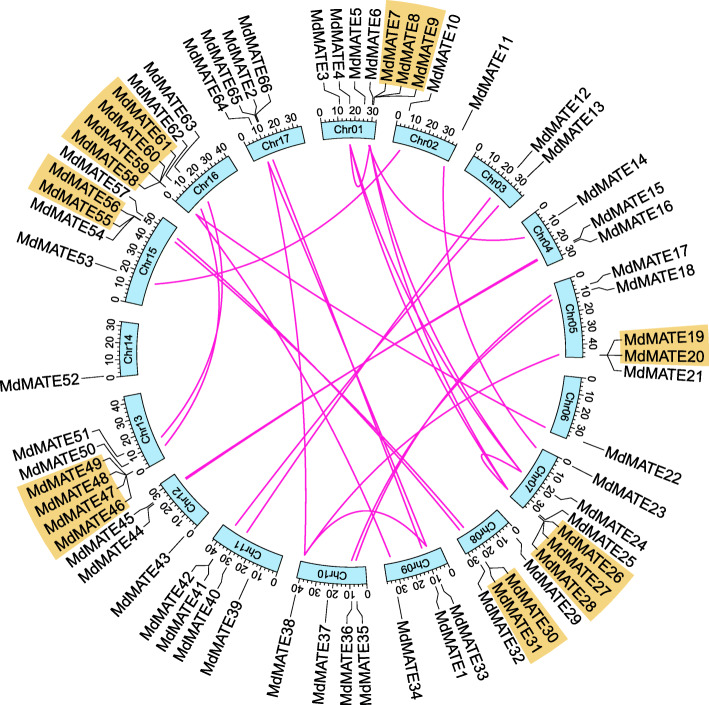
Chromosome distribution and collinear relations of *MdMATE* genes. The scale on the circle is in Mega bases. Gene IDs on the chromosomes indicate their physical positions. Red lines indicate segmental duplicated *MdMATE* gene pairs. Orange regions indicate tandem clusters

The distribution of *MdMATE* genes is uneven on the 17 chromosomes of apple genome (2n = 34) (Fig. 3). Chromosome 1 contains the highest number of *MdMATE* genes (7), whereas both chromosomes 6 and 14 contain only one gene. There is no significant correlation between chromosome length and *MdMATE* gene number (Spearman’s *ρ* = 0.317, *P* = 0.2157). Additionally, majority of these *MdMATE* genes are located on the chromosome arms, which are the regions with relative high recombination rate [29].

Gene duplications are considered to be one of the major driving forces in the evolution of genomes and expansions of the gene families [30, 31]. Whole genome duplication, segmental duplication and tandem duplication are the major causes of gene family expansion in plants [32]. We detected the duplicated events for *MdMATE* genes by MCscanX [33]. As shown in Fi gure 3, 26 gene pairs with 39 (59%) genes were identified as WGD/segmental duplication, while 20 (30%) tandem duplicated genes were identified within 7 tandem duplicated gene clusters (Additional file 1: Table S3). These results indicate that MATE family in apple expands mainly by gene duplications, with WGD/segmental duplication as the driving force.

### Synteny analysis of MATE family in green plants

**Fig. 4:**
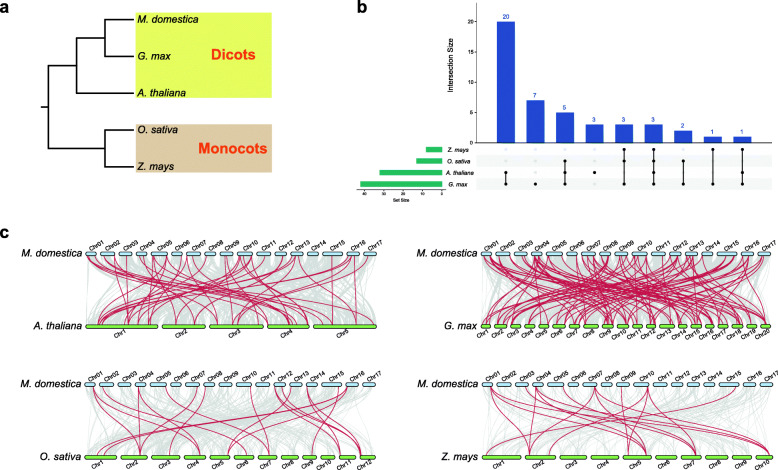
Synteny relationships of *MdMATE* genes with other four plant species. **A**. Species tree of apple and other four species. **B**. UpSet plot of collinear MATE genes between apple and other four species. Each vertical bar (dark blue) shows the number of *MdMATE* genes collinear with other species by the intersection matrix below it (a single dot in the matrix is a single species, 2 dots = 2 species, etc.). The number of genes in each species found to have collinear relationships with atleast one *MdMATE* genes is indicated by the horizontal bar (green) extending to the left. **C**. Synteny analysis of MATE genes between apple and other species. The red lines indicate the syntenic MATE gene pairs, while the gray lines in the background indicate the collinear blocks within apple and other plant genomes. The specie names with the prefixes ‘*M. domestica*’, ‘*A. thaliana*’, ‘*G. max*’, ‘*O. sativa*’ and ‘*Z. mays*’ indicate *Malus × domestica* Borkh., *A. thaliana*, *G. max*, *O. sativa* and *Z. mays*, respectively

As an integral membrane protein involved in a diverse array of functions, MATE family of transporters are abundant in plants [11]. We constructed four comparative syntenic maps of apple associated with four representative green plants to further investigate the phylogenetic mechanisms of MATE gene family in apple, including two dicots (*Arabidopsis* and soybean) and two monocots (maize and rice) (Fig. 4A). Among *MdMATE* genes, 68.2% (45 of 66 genes) were found associated with at least one collinear gene pair (Fig. 4B). A total of 42 *MdMATE* genes showed collinear relationship with 56 *GmMATE* genes in soybean, while 32 *MdMATE* genes showed collinear relationship with 26 *AtDTX* genes in *Arabidopsis*. In monocots, however, only 13 and 8 *MdMATE* genes collinear with 12 MATE genes in rice and 8 MATE genes in maize, respectively (Fig. 4C and Additional file 1: Table S4). Thus, the *MdMATE* genes show more collinear gene pairing with dicots than monocots.

For the collinear gene pairs between apple and soybean, which shows the most collinear relationships, 31 *MdMATE* genes are associated with two or more *GmMATE* genes. The *MdMATE14*, with 8 collinear *GmMATE* genes, is the gene with the most collinear relationships. In contrast, for *Arabidopsis*, most *MdMATE* genes associated with one or two *AtDTX* genes. Only *MdMATE38* associated three *AtDTX* genes. This might result from two whole genome duplication events in soybean [34]. Interestingly, 30.3% (20 of 66 genes) of *MdMATE* genes have collinear gene pairs in both *Arabidopsis* and soybean, but no collinear gene pairs in monocots (Fig. 4B). For example, *MdMATE1* collinear with *AtDTX41* and *GmMATE81*, but shows no collinear gene in maize or rice. This is similar with observation of WRKY family in pineapple in the sense that these MATE orthologous gene pairs appear after the divergence of dicotyledonous and monocotyledonous plants [31]. Notably, three *MdMATE* genes, *MdMATE14*, *MdMATE19*, and *MdMATE38*, have collinear relationships between apple and all of the other four species, indicating that these orthologous pairs are conserved and may already exist before the ancestral divergence. These collinear gene pairs between apple and other species may be valuable for elucidating the evolution of MATE genes in green plants.

To better understand the different selective constraints on MATE gene family, the Ka/Ks ratios of the MATE gene pairs within apple and between apple and the other four plants were calculated (Additional file 1: Table S4). All segmental and tandem duplicated *MdMATE* gene pairs and the orthologous MATE gene pairs had Ka/Ks < 1, suggesting that the MATE gene family in apple have experienced strong purifying selective pressure during the evolution.

### Expression patterns of *MdMATE* genes in different apple tissues/organs and developmental stages

**Fig. 5:**
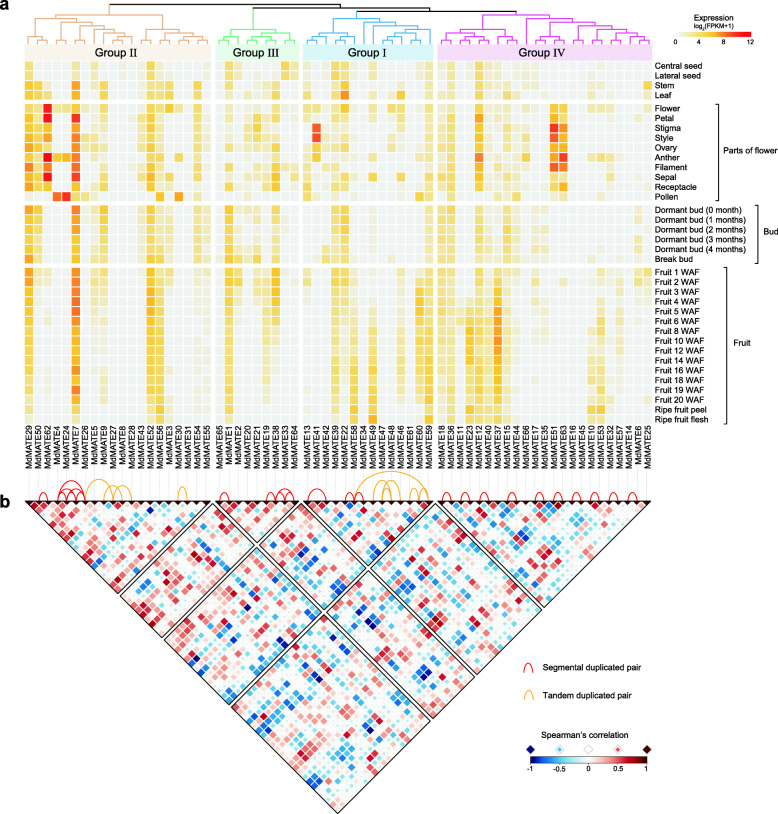
Expression profiles of *MdMATE* genes in different tissues/organs and developmental stages. **A**. Heatmap of the expression pattern in various tissues/organs and developmental stages. The name of tissues/organs and development stages are indicated on the right. WAF: week after full bloom. The FPKM values were transformed by log2. Phylogenetic tree on the top of heatmap is the same as Fig. 2A. **B**. Spearman correlation of gene expression pattern. Positive correlations are displayed in red and negative correlations in blue. Size and color intensity of the square are proportional to the value of Spearman’s ρ. Red and yellow lines on the top of correlation heatmap indicate segmental and tandem duplicated pairs, respectively

To dissect the expression patterns of *MdMATE* genes in various tissues/organs and developmental stages, a total of 36 expression profiles of 66 *MdMATE* genes were obtained from Apple Multi-Dimensional Omics Database (AppleMDO) [35] (Fig. 5A and Additional file 1: Table S5). The *MdMATE* genes with Fragments Per Kilobase per Million (FPKM) values less than 1 in all 36 expression profiles are considered to be barely expressed [4]. Thus, 11 genes are not expressed in all 36 tissues/organs and developmental stage profiles. The remaining 55 genes are expressed in at least one profile. Among them, *MdMATE1* is expressed in all developmental stages of fruit flesh and peel, while *MdMATE2* shows expression level declining from earlier to later developmental stages. This is consistent to the real-time RT-PCR results published by previously study [22].

Among the 55 expressed genes, *MdMATE7* and *MdMATE36* showed constitutive expression (FPKM > 1 in all tissues/organs and developmental stages). It suggests that these two genes may involve apple growth. Some gene expressions are tissue-specific. For example, *MdMATE24* has high expression in parts of flower, especially in pollen. *MdMATE23* expressed mainly in fruit and peel. While *MdMATE62* is highly expressed in parts of flower except for pollen, *MdMATE33* is highly expressed in central and lateral seed. The expression level of some genes gradually increases as the fruit matures (eg. *MdMATE23*, *MdMATE49* and *MdMATE58*) and some genes were show the other way (eg. *MdMATE2*, *MdMATE5* and *MdMATE38*). It suggests their putative roles during apple fruit development. These results show that most *MdMATE* genes have dynamic expressions in different tissues/organs or developmental stages. It can help us to explore the functional diversity of *MdMATE* genes in apple.

After gene duplication, the divergence of expression pattern of two copies is considered to be an important aspect in their functional differentiation [36, 37]. In the context of gene expression, it is straightforward to observe the function divergence after gene duplication [38]. Hence, correlation coefficient was calculated for the expression pattern of *MdMATE* gene pairs (Fig. 5B). The results show that genes in same group could differ considerably in their expression pattern, similar as before [39]. Among the 39 duplicated pairs, *MdMATE4*/*MdMATE26*, *MdMATE10*/*MdMATE53*, *MdMATE17*/*MdMATE35*, *MdMATE50*/*MdMATE62*, *MdMATE51*/*MdMATE63*, *MdMATE58*/*MdMATE59*, and *MdMATE59*/*MdMATE60* showed significant positive correlations (Spearman’s *ρ* > 0.60, *P* < 0.05), and only one gene pair *MdMATE22*/*MdMATE58* showed negative correlations (Spearman’s *ρ* = − 0.62, *P* < 0.05). As novel expression patterns may also only occur in specific organs, we further investigate the correlation of expression patterns in three organs (flower, bud and fruit) [40] (Additional file 2: Fig. S3). Intriguingly, some duplicated pairs showed organ-specific correlation (*MdMATE38*/*MdMATE64* in flower, *MdMATE13*/*MdMATE42* in bud and *MdMATE1*/*MdMATE65* in fruit) (Additional file 2: Fig. S3). Additionally, in fruit, genes in same group showed higher than overall correlation. Almost all members in group III (green) showed positive correlation. In summary, these results indicated that the functions of *MdMATE* genes in apple tissues/organs and developmental stages may be widely correlated and varied. These paralogs with tissue-specific expression pattern has long been regarded as a precursor of future evolution which may contribute to phenotypic variation [36, 41].

### *Cis*-acting regulatory element analysis

**Fig. 6:**
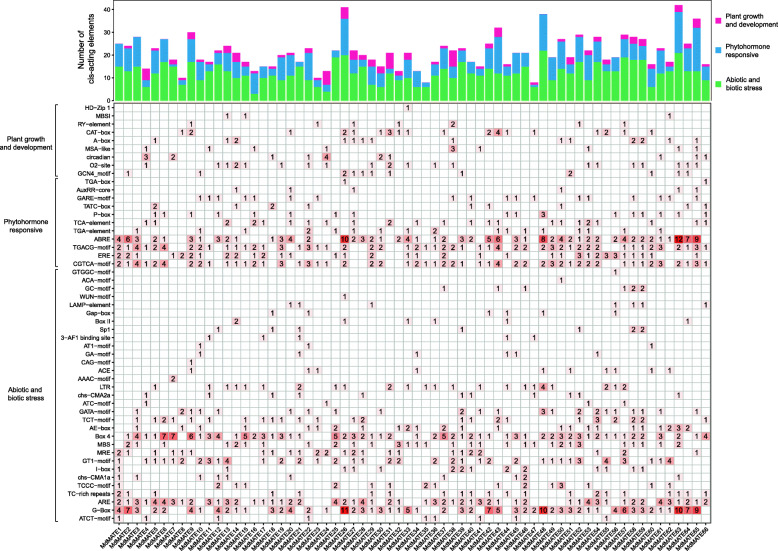
*Cis*-element analysis of 1.5 kb upstream region of *MdMATE* genes. The bars on the top represent the total number of *cis*-elements in each gene promoter region. Different colors represent different type of *cis*-elements. The color intensity and number in the cells indicated the numbers of *cis*-element in these *MdMATE* genes

Gene transcription in plants is regulated by the *cis*-acting regulatory elements and transcription factors [42].. To identify putative *cis*-acting elements in the promoter region, we scanned the 1.5 kb upstream regions of transcriptional start site (TSS). All the candidate *MdMATE* gene promoters possessed typical TATA and CAAT boxes which are the core *cis*-acting element in eukaryotic promoter and enhancer regions (Additional file 1: Table S6). Other *cis*-acting regulatory elements were grouped into three main types based on their functional annotation: plant growth and development, phytohormone responsive and abiotic and biotic stress (Fig. 6). The abiotic and biotic stress group had the most number of regulatory elements, such as G-box, MBS and LTR, which were responsive to light inducible, drought inducible and low-temperature stress, respectively. Many abiotic and biotic stress elements were observed in the promoter region of *MdMATE* genes, revealing that *MdMATE* genes play important roles on the stress response. The followed group is phytohormone responsive which has 11 elements, the ERE, ABRE, TGACG-motif and CGTCA-motif widely distributed among *MdMATE* members, which showed relatedness with Ethylene, Abscisic acid (ABA) and Methyl jasmonate (MeJA) responses, respectively. A total of nine *cis*-elements related to plant growth and development were also identified in some *MdMATE* gene promoter regions and the metabolism regulation element (O_2_-site) was found in 20 *MdMATE* genes. Various regulatory elements were identified, suggesting that the *MdMATE* genes play a crucial role in a wide range of biological processes in apple.

### Expression of *MdMATE* response to pathogen infection

**Fig. 7:**
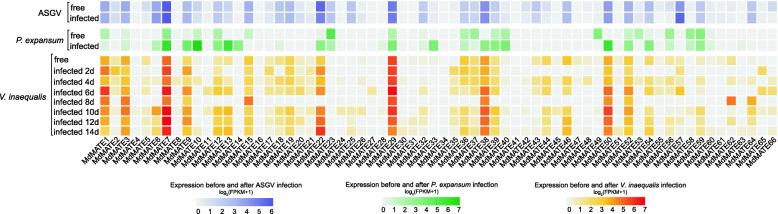
Heatmap of the expression profiles of *MdMATE* genes in different ASGV, *P. expansum* and *V. inaequalis* infection. The FPKM values were transformed by log2. Detailed values can be found in Additional file 1: Table S7

In apple and other fruits, the quality and yield can be greatly reduced in the presence of diseases [43]. An increasing body of evidence shows that the *MATE* genes are essential in conferring tolerance to abiotic and biotic stress factors [11, 21, 44].. To explore the role of *MdMATE* genes in response to disease, we analyzed the changes of transcription levels of *MdMATE* in response to *Apple stem grooving virus* (ASGV), *Penicillium expansum* (*P. expansum*) and *Venturia inaequalis* (*V. inaequalis*). These three pathogens affect the quality and yield of apples, cause a lot of economic losses and waste. In the control before and after the ASGV infection, the expression levels of *MdMATE57* showed up-regulated, while *MdMATE22* showed down-regulated (Fig. 7). After *P. expansum* infection, a total of 16 and 7 genes were up-regulated and down-regulated, respectively (Fig. 7). *MdMATE7*, *MdMATE10*, *MdMATE13* and *MdMATE50* barely expressed in *P. expansum* free, while their expression level in terms of FPKM increases to 94.76, 182.35, 65.64 and 82.61 after *P. expansum* infection, respectively (Additional file 1: Table S7). In contrast, *MdMATE23*, *MdMATE37* and *MdMATE49* showed dramatical decrease after *P. expansum* infection. Apple scab is the most serious disease of apple worldwide in terms of the economic cost of control [45, 46]. It is caused by *V. inaequalis*, a fungus that can cause the apple disease. Scab can be found in almost all areas in which apples are grown commercially. Here we analyzed the *MdMATE* gene expression levels of different stages after *V. inaequalis* infection (Fig. 7). Some genes showed up-regulated after infecting by *V. inaequalis* such as *MdMATE2*, *MdMATE7* and *MdMATE9*, whereas some genes showed down-regulated such as *MdMATE3*, *MdMATE15* and *MdMATE46*. The expression level of *MdMATE6*, *MdMATE59*, *MdMATE62* and *MdMATE64* increased after 8 days of infection. These results indicated the MATE gene family in apple have a diverse function in responses to biotic stress, especially for *P. expansum* infection.

## Discussion

Recently, the genome-wide analysis of MATE gene family in different species has been gradually carried out, suggesting their diverse function involved in stress responses, plant growth and transmembrane transport. In this study, we carried out a comprehensive analysis of MATE gene family in apple. We identified 66 *MdMATE* genes from the ‘Golden Delicious’ reference genome, including two genes that have been reported. We conducted phylogenetic, gene structure, motif pattern, synteny and evolutionary analysis. Besides, the expression patterns of *MdMATE* genes in different tissues/organs and developmental stages were also explored. The *cis*-element analysis shows that *MdMATE* genes are widely involved in stress response in apples. Hence, we also compared the changes in *MdMATE* gene expression before and after three different pathogen infections.

A total of 66 *MdMATE* genes were identified in apple. The MATE members in apple exceed those in *Arabidopsis* (56 members, ~ 135 Mb) [10], potato (48 members, ~ 674 Mb) [6], maize (49 members, ~ 2300 Mb) [15] and rice (45 members, ~ 380 Mb) [16], but family size is inferior to soybean (117 members, ~ 978 Mb) [17]. The number of *MATE* genes is not paralleled to the size of genome and there is no absolute correlation with genome size. However, in dicots, it was paralleled to the genome size and generally have more members than monocots. Further synteny analysis showed there are more collinear gene pairs between apple and dicots than monocots. We speculated that the expansion of MATE gene family mainly occurs during the evolution of dicots.

Phylogenetic analysis divides the *MdMATE* genes into four groups based on the classifications on *AtDTX* proteins (Fig. 1). In group I, the *MdMATE* lost five members, while group II, III and IV gained four, three and eight members, respectively. Group I contains 14 MdMATE transporters along with 19 AtDTX transporters. Among them, the *AtDTX1*, which is the first plant protein that has been shown to function as a detoxifying efflux carrier, can mediate the efflux of plant-derived or exogenous toxic compounds from the cytoplasm [10]. The *AtDTX14* and *AtDTX18* can export norfloxacin and prevent *Arabidopsis* colonization by *P. infestans*, respectively [47, 48]. The *AtDTX19* is expressed in root epidermal cells and necessary for protecting roots from toxic compounds in the soil [12]. Group II contain 20 MdMATE transporters along with 16 AtDTX transporters. Among them, the *AtDTX21* plays an important role in Atrazine detoxification [49]. The *AtDTX30*, *AtDTX31*, *AtDTX33* and *AtDTX35* control root hair development in *Arabidopsis* [50, 51]. Besides, the *AtDTX30* also involve in aluminum tolerance and indirectly modulated citrate exudation [52]. Group III contain 9 MdMATE transporters along with 6 MATE transporters from *Arabidopsis*. The *AtDTX41*/*TRANSPARENT TESTA 12* (*TT12*) had been functionally characterized that can mediates anthocyanin transport in vitro [13]. Its homologs in apple, *MdMATE1* and *MdMATE2*, are vacuolar flavonoid transporter which are active in proanthocyanidins accumulating cells of apple fruit [22]. In the two subgroups of group IV, the group IV-a contain 9 and 6 MATE transporters from apple and *Arabidopsis*, respectively. The *AtDTX42* can facilitate AI-activated citrate exudation independently from and along with *AtALMT1* to confer a full expression of *Arabidopsis* AI tolerance [53, 54]. The *AtDTX43* plays a major role in iron and zinc homeostasis in *Arabidopsis* and transports citrate [55]. The *AtDTX47* is related to the transport of salicylic acid or its precursor [56]. While 14 MdMATE transporters and 9 AtDTX transporters in the group IV-b. The *AtDTX48* is related to multiple functions, including organ initiation, iron homeostasis and hypocotyl cell elongation [57–59]. The *AtDTX50* can transport abscisic acid (ABA) and respond to drought conditions [18]. The function of *AtDTX51* is related to hypocotyl cell elongation same as *AtDTX48*, but additionally has the functions of regulates plant disease resistance and affects plant architecture [60, 61]. The *AtDTX51* and *AtDTX52* control senescence and iron homeostasis in plants [62, 63]. The *AtDTX54* and *AtDTX55* have a conserved function in the regulation of lateral organ initiation in plants [19]. The *AtDTX56* can repress a protein kinase that negatively regulates CO_2_-induced stomatal closing [64]. From the functions of these homologous AtDTX transporters in the subgroups of group IV, the function of group IV-a is mainly related to ion tolerance and in group IV-b, it is also related to developmental and disease resistance in addition to the functions similar as group IV-a. Overall, *Arabidopsis* MATE members in the same group have highly diverse functions, indicating MATE in apple may also have multitasking ability.

In group I, only four *AtDTX* genes (*AtDTX1*, *AtDTX14*, *AtDTX18* and *AtDTX19*) [10, 12, 47, 48] were functionally characterized but no duplicated *MdMATE* genes pairs clustered together with these *AtDTX* genes. However, in group IV, duplicated *MdMATE* genes pairs such as *MdMATE12*/*MdMATE40*, *MdMATE15*/*MdMATE44*, *MdMATE18*/*MdMATE36*, *MdMATE57*/*MdMATE32*, *MdMATE53*/*MdMATE10*, *MdMATE45*/*MdMATE16*, *MdMATE17*/*MdMATE35* and *MdMATE51*/*MdMATE63* were clustered together with *AtDTX42*, *AtDTX44*, *AtDTX46*, *AtDTX48*, *AtDTX51*, *AtDTX53*, *AtDTX54* and *AtDTX55*, respectively. Some of these *AtDTX* genes are involved in diverse mechanisms that are indispensable to plant growth and development: the *AtDTX42* is related with AI tolerance [53, 55]; the *AtDTX48*, *AtDTX51*, *AtDTX54* and *AtDTX55* all related to plant growth [19, 57–61]. The duplicated *MdMATE* gene pairs retained during the evolution process of apple may have similar functions with these neighbor *AtDTX* genes.

Different expression patterns of *MdMATE* genes were observed in various tissues/organs and developmental stages. It is worth noting that *MdMATE7* and *MdMATE36* are expressed in all samples in our study. These two genes may be important for maintenance of apple growth and development. Furthermore, expression correlation analysis reveals that *MdMATE* duplicates may follow different functional models (Additional file 1: Table S3). *MdMATE51*/*MdMATE63* have strong positive correlation (Spearman’s *ρ* = 0.878, *P* < 0.001) in flower. Both their expression levels were relatively high. The corresponding gene dosage increase may be beneficial for organism [38]. *MdMATE22*/*MdMATE58* show strong negative correlations (Spearman’s *ρ* = − 0.882, *P* < 0.001) in fruit developmental stages. In the early stages, *MdMATE22* expression level was higher than *MdMATE58*, but opposite in the late fruit development stage. This suggested that sub-functionalization probably occurred between the pair of genes [38, 65].

*Cis*-elements analysis further confirmed the versatility of MATE gene family and its major role in response to stress. By analyzing changes in the expression levels of *MdMATE* genes before and after infection of three pathogens that have a great impact on the quality and yield of apples, suggesting that *MdMATE* genes are involved in apple response to pathogen infection, especially for *P. expansum* infection. Notably, *MdMATE7* is involved in both infections by *P. expansum* and *V. inaequalis* (Fig. 7), and this gene is expressed in all tissue/organs. Thus, we speculated that the sustained expression of *MdMATE7* is important for apple growth and stress response. Additionally, the promoter regions of all *MdMATE* genes contain many stress response *cis*-elements and we further speculated that the other genes, which are not involved in the three pathogens infections has great potential for stress response.

## Conclusions

In this study, a total of 66 MATE (*MdMATE*) genes encoding MATE transporters were identified in the apple genome. We classified these *MdMATE* genes into four groups by phylogenetic analysis with MATE genes in *Arabidopsis*. Synteny analysis reveals that whole genome duplication and segmental duplication events played a major role in the expansion of *MATE* gene family in apple. *MdMATE* genes show diverse expression patterns in different tissues/organs and developmental stages. Analysis of *cis*-regulatory elements in *MdMATE* promoter regions indicates that the function of *MdMATE* gene is mainly related to stress response. Besides, the changes of gene expression levels upon different pathogen infections reveal that *MdMATE* genes are involved in biotic stress response. Our results provide insights for a more comprehensive understanding of the MATE gene family function in apple and provide valuable resources for apple disease resistance research.

## Methods

### Identification of MATE transporters in apple genome

A total of 56 MATE family members in *Arabidopsis* as previously reported [10] were download from The Arabidopsis Information Resource (TAIR) (https://www.arabidopsis.org/). A total of 101 apple putative MATE protein sequences were retrieved by BLASTP searches against the target apple proteome, GDDH13 v1.1 [23], using 56 *A.thaliana* MATE protein sequences as queries (E-value ≤10^− 7^) (Additional file 1: Table S1). To obtain more accurate MATE members in apple, manual filtering for the putative MATE protein sequences was performed as previously described [28]. Briefly, the putative MATE protein sequences were filtered by the presence of conserved MATE domain (Pfam: PF01554) using the HMMER (https://www.ebi.ac.uk/Tools/hmmer/search/hmmscan) [66], the Conserved Domain Database (CDD, https://www.ncbi.nlm.nih.gov/Structure/cdd/wrpsb.cgi) [67] and the Simple Modular Architecture Research Tool (SMART, http://smart.embl-heidelberg.de/smart/batch.pl) [68]. All these putative sequences were assessed against the expected features of the MATE transporters in plants (e.g., containing MatE domains, 8–12 transmembrane domains and classified as MATE_like superfamily) (Additional file 1: Table S1). Finally, a total of 66 apple MATE proteins were identified. Physical parameters such as theoretical isoelectric point (pI), molecular weight (MW) and instability index (II) were calculated by ProtParam (https://web.expasy.org/protparam/) [69]. The subcellular localization of the MATE proteins were predicted using WoLF PSORT [70].

### Chromosomal distribution and gene duplication analysis of MdMATE family

The physical location information of all 66 *MdMATE* genes were obtained from the apple genome annotation gff3 format file and visualize through Circlize package [71]. WGD/segmental and tandem duplication events were detected by MCScanX [72] with default parameters. Tandem clusters were defined as previously report: genes in a cluster need to be on the same chromosome and not more than one gene apart [73]. To exhibit the synteny relationship of the orthologous MATE genes in apple and other four species, MATE gene family members in *Arabidopsis*, soybean, maize and rice were obtained as the previous report [10, 15–17] and sequence files were download from Phytozome database (v12, https://phytozome.jgi.doe.gov/pz/portal.html) [74]. Species tree were obtained from TimeTree(http://www.timetree.org) [75]. Then MCScanX pipeline [33] was used to construct syntenic maps and visualization. Ka/Ks values between homologous were calculated by KaKs_Calculator 2.0 [76].

### Phylogenetic and gene structural analysis

All the 122 MATE protein sequences in apple and *Arabidopsis* were used to perform phylogenetic and structural analysis. Multiple sequence alignments were conducted by ClustalW in MEGA X with default parameters and ProteinWeightMatrix = BLOSUM [77]. The alignment result was then used to construct a phylogenetic tree based on the neighbor-joining (NJ) method of MEGA X, with the following setups: Equal input model and partial deletion (60%). For the maximum likelihood (ML) tree, JTT + F + G was used as the best model for ML tree constructing which calculated by ProtTest 3.4.2 [78], the ML tree was constructed by MEGA X. Both NJ and ML trees were conducted 1000 bootstrap replications.

Gene structure analysis was performed using the Gene Structure Display Server (GSDS) [79] with default setting. Motifs in MATE proteins were identified using MEME Suite [25] (version 5.1.1, http://meme-suite.org/index.html) with default setting: site distribution is zero or one occurrence per sequence (zoops), motif width is 5 to 50 and the maximum number of motifs was set at 12. We extract the 1.5 kb upstream region of *MdMATE* genes and upload to PlantCARE [80] database to detect *cis*-regulatory elements in the promoter regions.

### Expression pattern of *MdMATE* genes in different tissues and pathogen infection

We obtain a total of 48 expression profiles of 66 *MdMATE* genes from Apple Multi-Dimensional Omics Database (AppleMDO) [35]. These profiles including 36 tissues/organs and different development stage (central seed, lateral seed, stem, leaf, flower, petal, stigma, style, ovary, anther, filament, sepal, receptacle, pollen, four dormant bud stage, break bud, fourteen fruit developmental stages from 1 week after full-bloom (WAF1) to harvest (WAF20), ripe fruit peel and flesh and control data of three pathogens infection. In the database, all the RNA-seq data were already quality controlled and FPKM values can be extracted from the database. Expression heatmaps were made by Pheatmap package with pheatmap2 function [81]. Spearman correlation was conducted using the cor() function in R-4.0.2 (https://www.r-project.org/).

## Supplementary Information

**Additional file 1 Table S1**. Manual filtering of 101 putative MATE proteins. **Table S2**. Details of the 66 MdMATE genes in apple. **Table S3**. Duplicated gene pairs of MdMATE genes and expression pattern correlations. **Table S4**. Collinear gene pairs between MdMATE genes and MATE genes in other species. **Table S5**. FPKM of MdMATE genes in different tissues/organs and developmental stages. **Table S6**. Cis-elements of MdMATE genes. **Table S7**. FPKM of MdMATE genes with three pathogen infections.

**Additional file 2 Fig. S1**. The unrooted neighbor-joining phylogenetic tree of MATE family members in apple and Arabidopsis. The different colors indicate different groups (Group I in blue, Group II in orange, Group III in green and Group IV in pink). ‘MdMATE’ represents MATE members from apple, ‘AtDTX’ represents MATE members from Arabidopsis. Numbers on the nodes are bootstrap values in percentage (1000 replicates). **Fig. S2**. The conserved motifs among *MdMATE* proteins. **Fig. S3**. Spearman correlation of gene expression pattern in flower, bud and fruit. Positive correlations are displayed in red and negative correlations in blue color. Size and color intensity of the square are proportional to the Spearman’s *ρ*. Red and yellow lines on the top of correlation heatmap indicate segmental and tandem duplicated pairs, respectively.

## Data Availability

The reference genome were GDDH13 Version 1.1 and obtained from The Apple Genome and Epigenome (https://iris.angers.inra.fr/gddh13/the-apple-genome-downloads.html). All data analyzed during this study are included in this article and its Additional files. *MdMATE* gene names and details can be found in Additional file 1: Table S1 and S2. The Ka/Ks and spearman’s correlation coefficient of each gene pairs can be found in Additional file 1: Table S3 and Table S4. The FPKM value of *MdMATE* genes in different tissues/organs and pathogen infections can be found in Additional file 1: Table S5 and Table S7. The cis-elements annotations of *MdMATE* genes can be found in Additional file 1: Table S5.

## Abbreviations

ABA: Transport abscisic acid
ABC: ATP-binding cassette superfamily
ASGV: Apple stem grooving virus
FPKM: Fragments Perk Kilobase per Million
MATE: Multidrug and toxic compound extrusion
MFS: Major facilitator superfamily
MW: Molecular weight
NJ: Neighbor-joining
pI: Theoretical isoelectric point
RND: Resistance/nodulation/division family
SMR: Small gene multidrug resistance family
WGD: Whole genome duplication
