# Supplementary material for: Genome-wide identification, characterization and expression analysis of MATE family genes in apple (Malus × domestica Borkh)
Source: BMC Genomics. 2021 Aug 30;22:632. doi: 10.1186/s12864-021-07943-1 (PMC8406601; doi:10.1186/s12864-021-07943-1)
Supplement: Supplementary file 2 — Additional file 2 Fig. S1. The unrooted neighbor-joining phylogenetic tree of MATE family members in apple and Arabidopsis. The different colors indicate different groups (Group I in blue, Group II in orange, Group III in green and Group IV in pink). ‘MdMATE’ represents MATE members from apple, ‘AtDTX’ represents MATE members from Arabidopsis. Numbers on the nodes are bootstrap values in percentage (1000 replicates). Fig. S2. The conserved motifs among MdMATE proteins. Fig. S3. Spearman correlation of gene expression pattern in flower, bud and fruit. Positive correlations are displayed in red and negative correlations in blue color. Size and color intensity of the square are proportional to the Spearman’s ρ. Red and yellow lines on the top of correlation heatmap indicate segmental and tandem duplicated pairs, respectively. [file 12864_2021_7943_MOESM2_ESM.pdf]

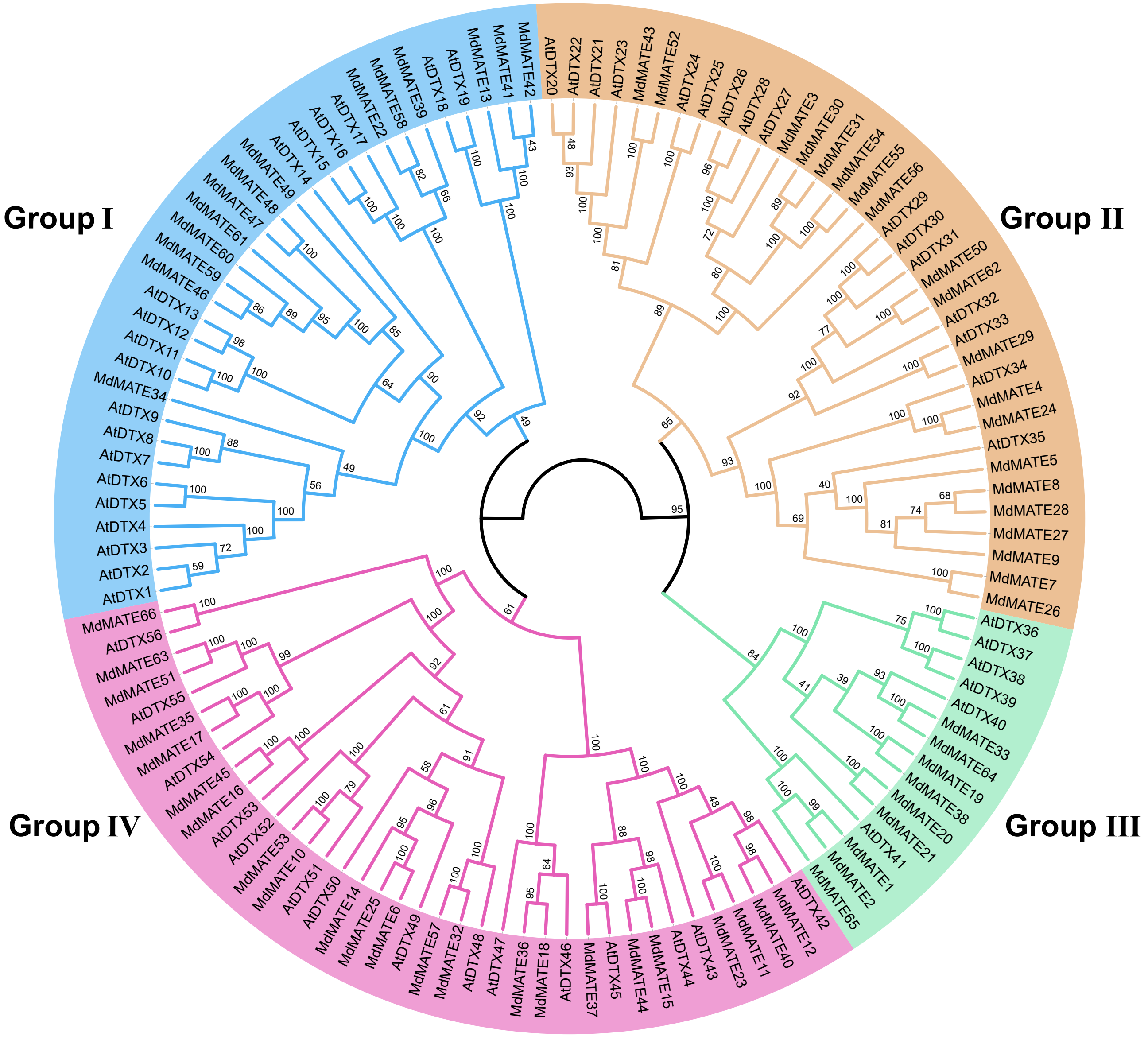

Figure S1. The unrooted neighbor-joining phylogenetic tree of MATE family members in apple and Arabidopsis. The different colors indicate different groups (Group I in blue, Group II in orange, Group III in green and Group IV in pink). 'MdMATE' represents MATE members from apple, 'AtDTX' represents MATE members from Arabidopsis. Numbers on the nodes are bootstrap values in percentage (1,000 replicates).

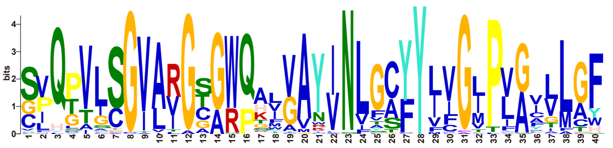

Motif 1: SVQPVLSGVARGSGWQALVAYINLGCYYJVGLPVGVLLGF

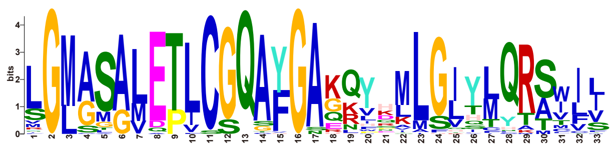

Motif 2: LGMASALETLCGQAYGAKQYHMLGIYLQRSWIL

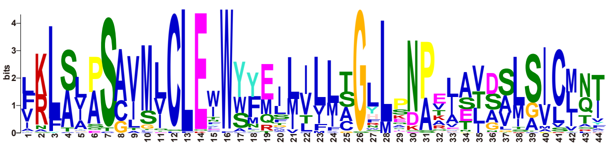

Motif 3: LKLSLPSAVMLCLEWWYYEILILLTGLLPNPEJAVDSLSICMNT

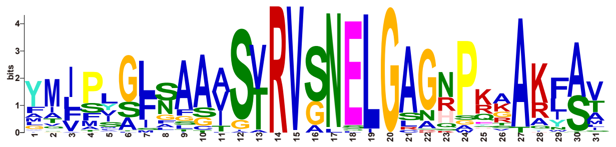

Motif 4: YMIPLGLSAAASVRVSNELGAGNPKAAKFAV

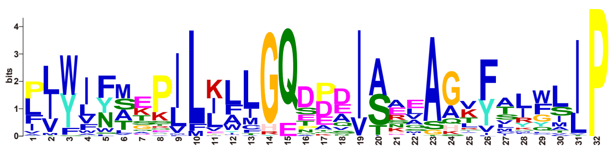

Motif 5: LLWIFMEPILKLLGQDPDIAAEAGKFAJWLIP

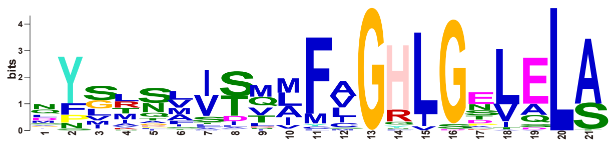

Motif 6: NYSLSLISMMFAGHLGELELA

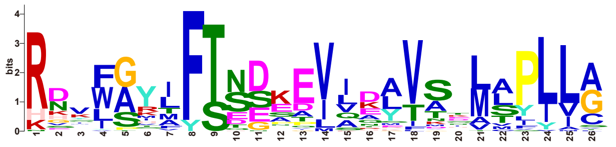

Motif 7: RBVFGYJFTNDKEVIDAVSTLAPLLA

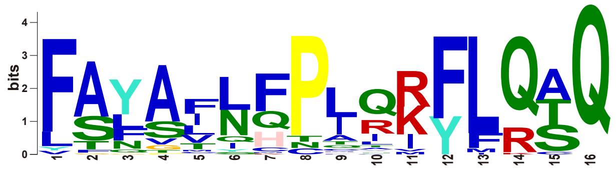

Motif 8: FAYAFLFPJQRFLQAQ

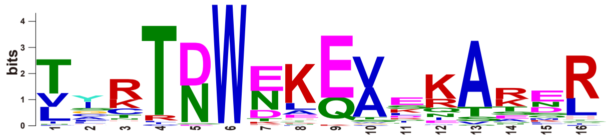

Motif 9: TYRTBWEKEVEKARER

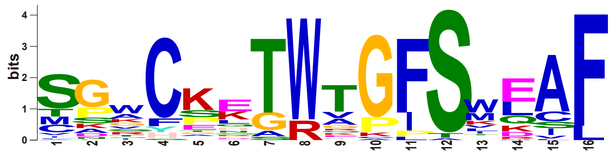

Motif 10: SGWCKETWTGFSWEAF

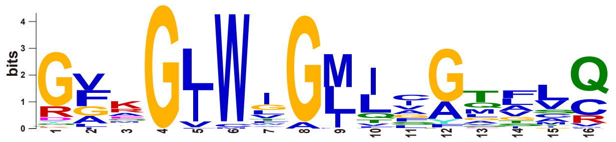

Motif 11: GVKGJWIGMJCGTFLQ

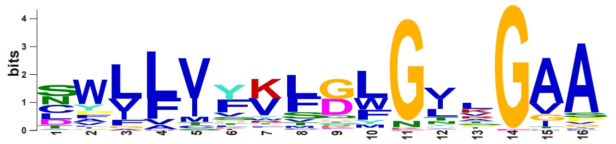

Motif 12: SWLLVYKLGLGVLGAA

Figure S2. The conserved motifs among *MdMATE* proteins.

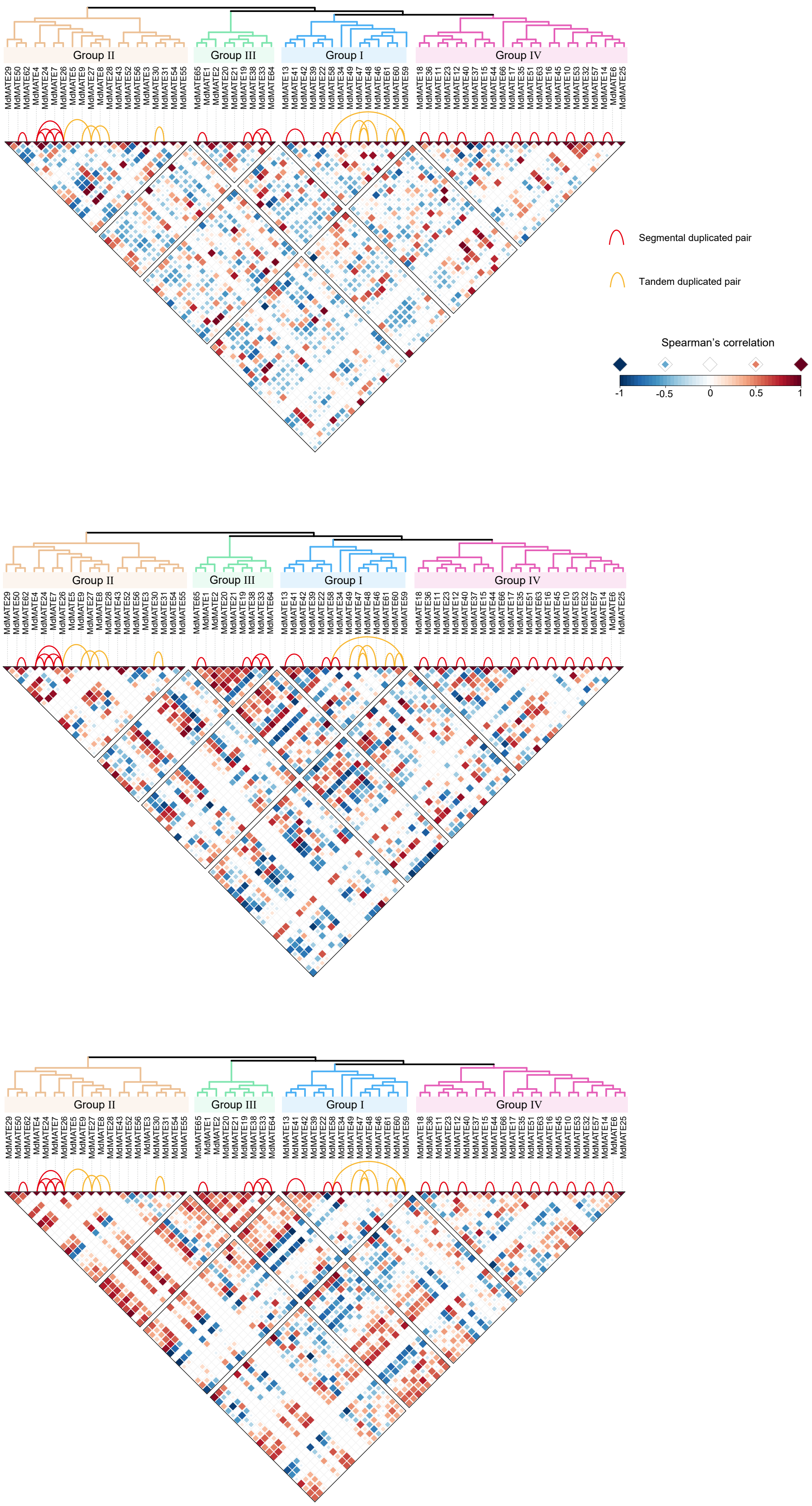

Figure S3. Spearman correlation of gene expression pattern in flower, bud and fruit. Positive correlations are displayed in red and negative correlations in blue color. Size and color intensity of the square are proportional to the Spearman's ρ. Red and yellow lines on the top of correlation heatmap indicate segmental and tandem duplicated pairs, respectively.
